# Enhancing the Performance of Medical Implant Communication Systems through Cooperative Diversity

**DOI:** 10.1155/2010/920704

**Published:** 2010-03-30

**Authors:** Barnabás Hegyi, János Levendovszky

**Affiliations:** ^1^Faculty of Information Technology, Pázmány Péter Catholic University, 1083 Budapest, Hungary; ^2^Department of Telecommunications, Budapest University of Technology and Economics, 1117 Budapest, Hungary

## Abstract

Battery-operated medical implants—such as pacemakers or cardioverter-defibrillators—have already been widely used in practical telemedicine and telecare applications. However, no solution has yet been found to mitigate the effect of the fading that the in-body to off-body communication channel is subject to. In this paper, we reveal and assess the potential of cooperative diversity to combat fading—hence to improve system performance—in medical implant communication systems. In the particular cooperative communication scenario we consider, multiple cooperating receiver units are installed across the room accommodating the patient with a medical implant inside his/her body. Our investigations have shown that the application of cooperative diversity is a promising approach to enhance the performance of medical implant communication systems in various aspects such as implant lifetime and communication link reliability.

## 1. Introduction

With the ever-growing development of information and communication technologies, novel types of services such as telemedicine and telecare [1] have become feasible. One important technological component of these remotely delivered services is obviously wireless communications, primarily radio communications.

In 2002, the European Telecommunications Standards Institute (ETSI) standardized the Medical Implants Communications System (MICS) [2], which is specified to be used by active medical implants communicating to each other or to an external controller in the UHF band (402–405 MHz). MICS shares the allocated spectrum with the Meteorological Aids Services (METAIDS), which is primarily used by weather balloons [3]. For this reason, MICS is limited to operate indoors only [1]. (Note that a similar standard was issued for the United States by the Federal Communication Commission (FCC) in 1999 [4].)

A good example of a telemedicine system operating in the MICS band is the Biotronik Home Monitoring system [5] (or Medtronic's Conexus Wireless Telemetry [6]). In this particular system, the data (trend, event, etc.) transmitted by the implanted pacemaker or cardioverter-defibrillator (operated by a nonrechargeable battery) is received by an exterior device, which then automatically forwards it to Biotronik's Service Centre via the cellular telephone system (GSM).

The communication between a body-implanted device and an off-body transceiver is treated by only a relatively few papers in the literature on a link level. One such treatment is provided in [7, 8], where the performance of a traditional (noncooperative) radio link between a medical implant and a single base station—both located in the same hospital appointment room—is considered. The results of another related study are presented in [9], where a transmit diversity scheme was proposed to decrease the transmission power of the implant using interconnected transceivers inside the human body. (This solution obviously implies a rather invasive medical intervention as the transmitters need to be implanted with a considerable distance between them in order to achieve a substantial diversity gain.) However, except for our present attempt to do so, no effort to date in the literature has been made to unveil or to assess the potential of cooperative diversity [10, 11] to combat fading—hence to improve system performance—in medical implant communication systems. (Remark that concerning the different fading effects the in-body to off-body propagation channel is subject to, multipath fading could be alleviated with a single receiver of reasonable size. The mitigation of shadow fading (see Section 4.3) through traditional spatial diversity would, however, require an impractically large antenna separation, and therefore it is not an option when designing an implant communication system.)

The remainder of the paper is organized as follows. Section 2 presents the applications scenario. In Section 3, the topology model is described along with the energy consumption model of the communication nodes. The radio propagation model is set up based on the results in the literature in Section 4. Section 5 details the different stages of the cooperative communication procedure under investigation, while the numerical results are presented in Section 6. Section 7 concludes the paper.

## 2. Application Scenario

**Figure 1:**
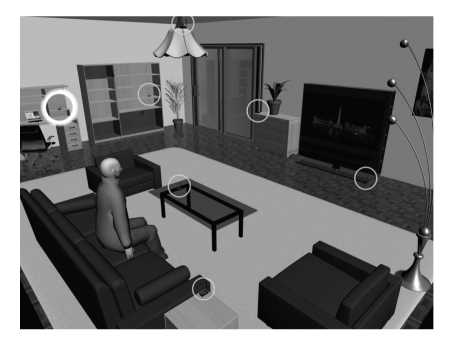
The room accommodating the patient. The CRUs and the G-CRU installed across the room are highlighted by the thin circles and the thick circle, respectively. The packet transmitted by the implant is received by multiple CRUs, which then cooperatively make the decision on the implant packet.

In our particular application scenario, we consider a single room accommodating a patient with a medical implant inside his/her body (Figure 1). The room may be located, as an example, at the patient's home or work place or at the hospital. The implant intends to communicate its sensed data to the outside world at regular intervals. In the case of the traditional, noncooperative communication approach, the data is received by the only receiver unit in the room, which is placed in the immediate proximity of the patient—for example, at the bedside table, at the desk, or next to the armchair depending on the activity the patient is performing. Contrary to that, for the cooperative communication scenario, the packet transmitted by the implant is received by multiple battery-operated, wireless cooperating receiver units (CRUs) installed across the room (Figure 1). (In addition to the aforesaid role, these nodes may also serve as a part of a larger ambient sensor or perhaps some ad hoc network in the building incorporating the room in question [12].) After that, a couple of the CRUs are selected for cooperation. Finally, these CRUs relay their packet to the gateway CRU (G-CRU) in the room, which then makes the final decision on the implant packet and forwards it to the service centre through, for example, the internet or the 3G network. (Please remark that when the patient stays outdoors, he/she uses a single receiver unit worn on the belt.)

It is apparent that by applying multiple receiver units in different locations of the room, the required transmission power of the implant can be reduced under a given reliability constraint. This reduction in transmission power can be converted into lifetime gain, which stands in the main focus of attention in this paper. Please note, however, that—for a fixed implant transmission power—the above described cooperative communication scheme offers the alternative benefit of a more reliable and a more spectrum efficient (faster) communication between the implant and the outside world. Furthermore, it is easy to see that the procedure makes it possible for the implant to transmit longer (more data) and more frequently under a given energy (lifetime) constraint. The choice between the possible benefits always depends on the specific application at hand.

Though the various benefits of the above described cooperative diversity scheme are easy to see, its actual gains are more difficult to predict. The aim of the present investigations is to quantify these gains, that is, determine whether the above solution implies a considerable or only a marginal improvement.

## 3. Topology and Energy Consumption Model

In this section, the topology and power consumption models applied in the forthcoming investigations are described in detail.

### 3.1. Topology Model

In order to simplify our investigations, we consider a two-dimensional (2D) spatial model in terms of the location of the communicating parties in the room. In other words, the implant and the CRUs are assumed to lie in the very same horizontal plane of the room.

Since our purpose is to evaluate the potential of cooperative diversity to prolong the lifespan of medical implants in general, we do not consider a specific spatial arrangement of the CRUs in the room. Instead, the positions of the CRUs are regarded as random variables; in particular, they are assumed to be distributed according to a (two-dimensional) homogeneous Poisson distribution. In accordance with this assumption, the number of CRUs in the room (*N*) is Poisson distributed with parameter *ρ* *A*, *A* being the area of the room and *ρ* the spatial (area) density of the CRUs. (Note that we assume that *ρ* *A* is high enough for the event *N* = 0 to have a negligible probability.) The locations of the CRUs (**r**_*i*_, *i* = 1,…, *N*) are then independent, identically distributed random variables that have a uniform distribution over the area of the room. Once again in order to investigate the problem in general, the G-CRU is chosen randomly from among the CRUs.

In order to take the mobility of the patient into account, we consider also the location (**r**) and orientation (**r**_0_) of the implant/patient as random variables. (The orientation of the patient is identical to a designated direction of the body, for instance, the direction the chest is facing.) We assume that **r** follows a uniform distribution over the area of the room, while **r**_0_ is assumed to be uniformly distributed over the unit circle.

The distances between the implant and CRU*_i_* and that between CRU*_i_* and CRU*_j_* are expressed as

(1) $$\begin{array}{c} d_{\text{I},\text{CR}{\text{U}}_{i}}=\left|r_{i}-r\right|,\\ d_{\text{CR}{\text{U}}_{i},\text{CR}{\text{U}}_{j}}=\left|r_{i}-r_{j}\right|, \end{array}$$

respectively. Finally, the orientation of CRU_*i*_ relative to the implant (*ϕ*_*i*_) is given by

(2) $$\phi_{i}=∠\left(r_{i}-r,r_{0}\right).$$

Please remark that the above topology model can easily be extended into three dimensions (3D). In that case, *N* is Poisson distributed with parameter *ρ* *V* with *ρ* and *V* being the spatial (volume) density of the CRUs and the volume of the room, respectively. In addition, the locations of the CRUs and that of the implant follow a uniform distribution over the volume of the room, whereas the orientation of the implant is distributed uniformly over the unit sphere.

### 3.2. Energy Consumption Model

We apply the power consumption model used in [13], which takes into account not only circuit power consumption but also the losses of the radio frequency (RF) power amplifier (PA). The power consumed at transmission (*P*_t_) is expressed as

(3) $$P_{\text{t}}\left(P_{\mathrm{tr}\;}\right)=\eta_{\text{PA}}^{-1}P_{\mathrm{tr}\;}+P_{\text{circ},\text{t}},$$

where *η*_PA_ is the efficiency of the RF PA of the transceiver, *P*_tr _ is the transmission power, while *P*_circ,t_ is the circuit power consumption at transmission.

Several low-power UHF transceiver designs for biomedical applications have recently been reported in the literature [14–18]. In order to make the analysis as realistic as possible, we are going to assume *P*_circ,t_ and *η*_PA_ values similar to those published in these papers for the implanted device in our simulations. (For specific values, please refer to Table 1).

## 4. Radio Propagation Model

In this section, the radio propagation model to be used throughout the performance analysis is set up. Based on the achievements in the literature on radio wave propagation from medical implants, we provide a plausible statistical model of the propagation channel between the implant and the CRUs (and that between the CRUs). The establishment of such statistical model is essential when assessing the potential of cooperative diversity to extend the lifetime of medical implants.

### 4.1. Multipath Fading

Johansson et al. measured the path loss between two external copolarized dipole antennas for the MICS band in an indoor environment [8, 19]. The measured path loss was compared to free space loss and its large-scale mean was found to be in good agreement with that. As a consequence, we are going to approximate the large-scale mean of the path loss with free space loss in our calculations.

Regarding the dispersiveness of the channel, multipath fading is considered to be flat [8, 19] (narrow band channel model), while concerning the time-variance of the channel, we assume block-fading, that is, the fading is regarded to be slow enough for the transmitted symbols within a single period of the communication cycle to experience the same channel gains. The latter assumption is reasonable as for a carrier frequency of 403.5 MHz and a mobility of 1 ms^−1^—corresponding to walking people—the coherence time of the channel is on the order of 100 ms. (Note that the former assumption is supported by the fact that the symbol time—corresponding to the maximum bandwidth of an MICS session (300 kHz) —is about two orders of magnitude higher than it takes the radio waves to travel across the room. Hence, in order for intersymbol interference to occur, a wave of nonnegligible power reflected off the walls around hundred times would be required.)

In order to incorporate multipath fading into our model, we assume Rice-fading [20] in the room as it is presumable that there is always a line-of-sight (LOS) multipath component (MPC) among the MPCs propagating between the implant and the CRUs. The *K*_r_ parameter of Rice-fading, called Rice-factor, is defined as the ratio of the power of the LOS MPC and that of the diffuse MPCs. Based on the excess loss values presented in [8], we estimated *K*_r_ to have a value of around 0 dB.

### 4.2. Polarization Mismatch

The direction-dependence of the polarization of radio waves transmitted by body-implanted devices in the MICS band was calculated and measured by Johansson [8] and Scanlon et al. [21], respectively. The calculations were carried out using the finite-difference time-domain (FDTD) method and employing homogeneous [8] or semisegmented (inhomogeneous) [21] numerical phantoms, whereas the measurements were performed applying a female subject [21]. In all cases, the polarization was found to show significant variations over the different directions of space.

Nevertheless, none of the above studies has given an analytical or even a statistical description of these variations. For this reasons, we propose the following simple probabilistic model for the (far-field) polarization of the wave transmitted towards CRU*_i_*:

(4) $$\begin{array}{c} p_{\vartheta ,i}^{\text{t}}=\cos\;\beta_{i},\\ p_{\varphi ,i}^{\text{t}}=\sin\beta_{i}\cdot e^{j\gamma_{i}}, \end{array}$$

where *β*_*i*_ and *γ*_*i*_ (*i* = 1,…, *N*) are independent random variables that are uniformly distributed over the interval [0,2*π*], while *p*_*ϑ*,*i*_^t^ and *p*_*φ*,*i*_^t^ are the components of the polarization in the spherical polar coordinate system centered at the implant with coordinates *ϑ* and *φ*, *ϑ* = 0 lying in the vertical direction. Differently from the other parts of the paper, *j* denotes the imaginary unit in this subsection. (Note that the polarization vector described in (4) is a random unit vector with a random phase difference between its components.) The above conditions also imply the assumption that the polarization of the waves transmitted towards the different CRUs is statistically independent.

According to the results presented in [8, 19], we assume in our calculations that the channel between the implant (including the body) and the CRUs does not alter the polarization.

When evaluating the effect of the mismatch between the polarization of the incident wave and that of the receiver antenna, the polarization of the receiver antenna—similarly to the polarization of the impinging wave—can be described by a unit vector. As an example, the polarization of a linearly polarized antenna—polarized in the *ϑ* direction—is represented by the unit vector

(5) $$\begin{array}{c} p_{\vartheta }^{\text{r},\text{lin}}=1,\\ p_{\varphi }^{\text{r},\text{lin}}=0, \end{array}$$

whereas the polarization of a right-circularly polarized antenna is described by the unit vector

(6) $$\begin{array}{c} p_{\vartheta }^{\text{r},\text{circ}}=\frac{1}{\sqrt{2}},\\ p_{\varphi }^{\text{r},\text{circ}}=j\frac{1}{\sqrt{2}}. \end{array}$$

We are going to apply these antenna types for the CRUs in our calculations.

Concerning the multipath fading the different polarization components are subject to, we assume in our model that the Rice-fading coefficients for the *ϑ*- and *φ*-components of the wave radiated towards CRU*_i_* (*a*_*ϑ*,*i*_^R^ and *a*_*φ*,*i*_^R^) are statistically independent [20]. These are given by

(7) $$\begin{array}{c} a_{\vartheta ,i}^{\text{R}}=\frac{1}{\sqrt{2\left(K_{\text{r}}+1\right)}}{\left|\sqrt{2K_{\text{r}}}+x_{\vartheta ,i}+jy_{\vartheta ,i}\right|}^{2},\\ a_{\varphi ,i}^{\text{R}}=\frac{1}{\sqrt{2\left(K_{\text{r}}+1\right)}}{\left|\sqrt{2K_{\text{r}}}+x_{\varphi ,i}+jy_{\varphi ,i}\right|}^{2}, \end{array}$$

where *x*_*ϑ*,*i*_, *y*_*ϑ*,*i*_, *x*_*φ*,*i*_, and *y*_*φ*,*i*_ (*i* = 1,…, *N*) are independent, standard Gaussian random variables. The conditions above also imply that the fading the different implant-to-CRU channels are exposed to is statistically independent. Note that this is a reasonable assumption as the fading dips investigated in [8, 19] were measured to be approximately 40 cm apart from each other.

### 4.3. Radiation Efficiency and Radiation Pattern

The radiation efficiency (*η*) of MICS-band wireless implants was calculated [8, 21] and measured [21] in several papers in the literature. The values obtained were approximately between −20 dB and −30 dB. (In other words, only between 0.1% and 1% of the total power radiated by the implant reaches the outside world, while the rest of it is simply absorbed by the body.) According to these results, we are going to apply similar radiation efficiency values in our investigations. In the above mentioned studies, also the radiation pattern of body-implanted transmitters was simulated and measured and the results revealed considerable variations in the radiation pattern. Moreover, the changes in body posture (e.g., arm movement) were found to cause significant alterations in the antenna pattern as well [8, 22]. The latter effects can be interpreted as one type of shadow fading [1].

**Figure 2:**
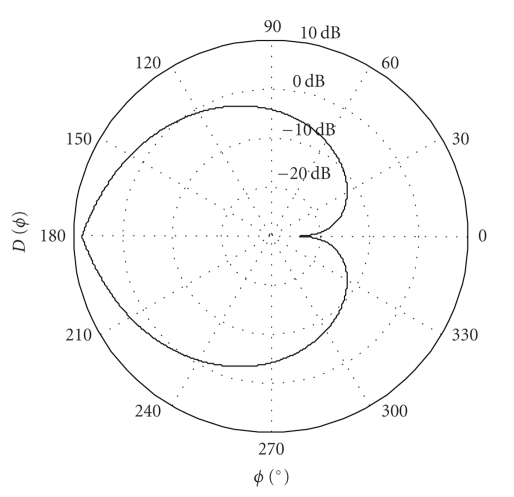
The assumed directivity pattern (*D*(*ϕ*)) of the implant. The pattern has the property that if we consider a random direction that follows a uniform distribution, then the corresponding directivity pattern value is distributed according to a (truncated) log-normal distribution.

Nonetheless, no analytical or detailed statistical description of either the radiation pattern or that of the impact of body posture has been provided by these studies. Variations due to shadowing effects show a log-normal distribution in most of the practical cases [20]. Moreover, when measuring the path loss from a physical numerical phantom filled with animal organs, the variations of the path loss around its mean were also attributed to shadowing effects by Alomainy et al. [23]. The measured path loss samples showed a log-normal distribution. As a consequence of these findings, we propose to approximate the variations in the radiation pattern and those due to the changes in body posture by the following axially symmetric 2D directivity pattern (Figure 2):

(8) $$D\left(\phi \right)={\left(\int_{0}^{2\pi }\tilde{D}\left(\phi \right)d\phi \right)}^{-1}\tilde{D}\left(\phi \right),$$

where

(9) $$\begin{array}{c} {\tilde{D}}^{\left[dB\right]}\left(\phi \right)=\{\begin{array}{cc} F^{-1}\left(\pi^{-1}\phi \right) & \text{if}\;0\leq \phi \leq \pi ,\\ F^{-1}\left(2-\pi^{-1}\phi \right) & \text{otherwise}, \end{array}\\ F\left(u\right)={\left(\int_{L}^{H}e^{-t^{2}/2\sigma }dt\right)}^{-1}\int_{L}^{u}e^{-t^{2}/2\sigma }dt,\;L\leq u\leq H. \end{array}$$

This pattern has the property that if we consider a random direction that follows a uniform distribution, then the corresponding directivity pattern value is distributed according to a truncated log-normal distribution. We truncate the original distribution at both ends in order to restrict its—otherwise infinite—dynamic range to those calculated in [7, 8, 22] and measured in [21]. Based on the margin and excess loss calculated in [7, 8, 22], we estimated the untruncated distribution to have a standard deviation of around *σ* = 9 dB, while the value of variables *L* and *H*—that is, the truncation limits—was estimated at −21 dB and 11.5 dB, respectively. (The normalization in the expression of *D*(*ϕ*) (8) is necessary in order for the directivity pattern (*D*(*ϕ*)) to have a unit mean. This normalization also implies the (optimistic) assumption that all of the emitted power of the implant is concentrated in the horizontal plane in question.)

For the 3D case, a directivity pattern with same properties can be obtained if we rotate the above described 2D pattern around the *φ* = 0 axis and then normalize it to have a unit mean with respect to *φ* and *ϑ*.

### 4.4. The Resulting Propagation Model

Now the only thing that remains is to piece together our propagation model in the following two equations, which concern the implant-to-CRU_*i*_ and the CRU_*i*_-to-CRU_*j*_ channels gains (*h*_I,CRU_*i*__ and *h*_CRU_*i*_,CRU_*j*__), respectively, (*i*, *j* = 1,…, *N*).

(10) $$\begin{array}{c} {\left|h_{\text{I},\text{CR}{\text{U}}_{i}}\right|}^{2}={\left(\frac{4\pi \lambda }{d_{\text{I},\text{CR}{\text{U}}_{i}}}\right)}^{2}{\left|a_{\vartheta ,i}^{\text{R}}p_{\vartheta ,i}^{\text{t}}p_{\vartheta ,i}^{\text{r}}+a_{\varphi ,i}^{\text{R}}p_{\varphi ,i}^{\text{t}}p_{\varphi ,i}^{\text{r}}\right|}^{2}\eta D\left(\phi_{i}\right),\\ {\left|h_{\text{CR}{\text{U}}_{i},\text{CR}{\text{U}}_{j}}\right|}^{2}={\left(\frac{4\pi \lambda }{d_{\text{CR}{\text{U}}_{i},\text{CR}{\text{U}}_{j}}}\right)}^{2}{\left|a_{\vartheta ,ij}^{\text{R}}p_{\vartheta ,i}^{\text{r}}p_{\vartheta ,j}^{\text{r}}+a_{\vartheta ,ij}^{\text{R}}p_{\varphi ,i}^{\text{r}}p_{\varphi ,j}^{\text{r}}\right|}^{2}, \end{array}$$

where the first factor in the equations is the expression of free space loss with *λ* being the wavelength in air. Variables *p*_*ϑ*,*i*_^r^ and *p*_*φ*,*i*_^r^ are the *ϑ*- and *φ*-components of the unit vector describing the polarization of the antenna of CRU*_i_*, respectively. We implicitly assumed in (10) that the CRUs are equipped with isotropic and lossless antennas and that the CRU*_i_*-to-CRU*_j_* channels are subject to the same type of multipath fading as the implant-to-CRU*_i_* channels. The fading factors for the former channel are denoted by *a*_*ϑ*,*ij*_^R^ and *a*_*φ*,*ij*_^R^.

The propagation model set up in this section is obviously just an approximation. This—among other things—is due to the fact that it has been put together based on the results of some studies that treated the different aspects of the propagation channel separately. We had to rely on this solution since—to the best of our knowledge—no paper to date investigates the properties of narrow band in-body to off-body propagation channels in an indoor environment directly and as a whole [9]. As an example, we assumed in our model that all of the power received at a given CRU originates from a single wave coming from the direction of the implant [8] and it is scattered only in the close vicinity of the CRU. This is not necessarily true in practice as waves of nonnegligible power that are reflected from relatively distant walls may also arrive at the CRU. Another limitation of the model is the assumption that even the closest CRUs are in the far field of the implant-body complex. (To the authors' knowledge, no information has yet been published on where exactly the border of the near and far field of a wireless implant lies. Nevertheless, it is noteworthy that in [7], a receiver placed at a distance of 1.64 m from an implant radiating in the MICS band is assumed to be in the far-field of the transmitter. As we will see later on, the mean distance of the patient and the closest CRU fall in the same order of magnitude in the forthcoming simulations.) However, the establishment of a more accurate statistical channel model—either by FDTD simulations or by measurements—requires a considerable amount of time and effort, and therefore it is out of the scope of this paper.

## 5. Cooperative Communication Scheme

In this section, the different stages of the cooperative communication scheme applied in our investigations are described. In addition, the definition of lifetime gain is also presented in detail.

The outline of the cooperative communication procedure is as follows. At the beginning of the procedure, the packet transmitted by the implant—containing the sensed data—is received by all the CRUs in the room. After that the relay selection stage takes place, during which the CRUs that are the most suitable for forwarding the implant packet to the G-CRUs are selected. In the course of the next stage, that is, the power allocation stage, the transmission power level of the relays is determined. Finally, the selected CRUs relay their packet to the G-CRU, which then optimally combines the different versions of the implant packet including the one that is directly received.

As our goal is mainly to draw attention to the possible advantages of cooperative diversity in medical implant communications, we consider only the simple case when the implant uses the same transmission power level at each and every transmission. The more complex problem of implant transmission power control is omitted in the paper.

### 5.1. Relaying Stage

There are a number of relaying schemes treated in the literature of cooperative communications. In our investigations, we apply the relaying method termed as amplify-and-forward (e.g., [24]), while in terms of medium access, we choose to employ the so-called repetition based relaying (e.g., [24]). In other words, we consider the case when the relays (i.e., the nongateway CRUs) amplify and retransmit their received analogue signal to the destination (i.e., to the G-CRU) one after the other.

Since the CRUs—with the exception of the G-CRU—are also assumed to be battery-operated, the power consumption of these nodes might also be of importance. As a consequence, we introduce a limit on the total transmission power of the relaying CRUs denoted by *P*_CRU_. Furthermore, in order to enhance the efficiency of transmission power utilization, we also treat the problem of optimal power allocation (among the relays) in our investigations.

The different versions of the implant packet are optimally combined at the G-CRU according to maximum ratio combining [20]. The problem of optimal (transmission) power allocation among the relays, though in a somewhat different context, was treated in [25]. According to this study, the expression of the resultant signal-to-noise ratio (SNR) of the combined relay packet—under the assumption of optimal power allocation—is given by

(11) $${\text{SNR}}_{\max\;}=\overset{n_{1}}{\underset{i=1}{\sum }}\frac{B_{\delta_{i}}}{C_{\delta_{i}}}-{\left(\overset{n_{1}}{\underset{i=1}{\sum }}\frac{\sqrt{B_{\delta_{i}}}}{C_{\delta_{i}}}\right)}^{2}{\left(1+\overset{n_{1}}{\underset{i=1}{\sum }}C_{\delta_{i}}^{-1}\right)}^{-1}$$

with

(12) $$\begin{array}{c} B_{i}=C_{i}\cdot \frac{P_{\text{I}}}{\sigma_{\text{n}}^{2}}{\left|h_{\text{I},\text{CR}{\text{U}}_{i}}\right|}^{2},\\ C_{i}=\frac{P_{\text{CRU}}{\left|h_{\text{CR}{\text{U}}_{i},\text{G}-\text{CRU}}\right|}^{2}}{P_{\text{I}}{\left|h_{\text{I},\text{CR}{\text{U}}_{i}}\right|}^{2}+\sigma_{\text{n}}^{2}}, \end{array}$$

where *n* is the (deterministic) number of selected relays, *P*_I_ is the transmission power of the implant, and *σ*_n_^2^ is the variance of the receiver noise. Parameter *n*_1_ in (11) is the largest integer that

(13) $$\begin{array}{c} \sqrt{B_{\delta_{n_{1}}}}g\left(n_{1}\right)>1,\;1\leq n_{1}\leq n,\\ g\left(k\right)={\left(\overset{k}{\underset{j=1}{\sum }}C_{\delta_{j}}^{-1}\sqrt{B_{\delta_{j}}}\right)}^{-1}\left(1+\overset{k}{\underset{j=1}{\sum }}C_{\delta_{j}}^{-1}\right),\;1\leq k\leq ,\\ \left\{\delta_{1},\ldots ,\delta_{n}\right\}\equiv \left\{1,\ldots ,n\right\} \end{array}$$

such that

(14) $$B_{\delta_{1}}\geq B_{\delta_{2}}\geq \cdots \geq B_{\delta_{n}}.$$

It is easy to see that, for the hypothetic case when *P*_CRU_ converges to infinity (CRUs with unlimited transmission power), the resultant SNR of the combined relay packet is given by

(15) $$\underset{P_{\text{CRU}}\to \infty }{\lim\;}{\text{SNR}}_{\max\;}=\overset{n}{\underset{i=1}{\sum }}\frac{B_{i}}{C_{i}}=\overset{n}{\underset{i=1}{\sum }}\frac{P_{\text{I}}}{\sigma_{\text{n}}^{2}}{\left|h_{\text{I},\text{CR}{\text{U}}_{i}}\right|}^{2}.$$

The expression behind the summation sign in (15) is the SNR of the implant-to-CRU_*i*_ channel (SNR_I,CRU_*i*__) and will henceforth be referred to as the received SNR of CRU_*i*_.

The combined relay packet and the packet that is received directly are optimally combined by the G-CRU according to MRC. Thus the resultant SNR of the packet to be decoded by the G-CRU is expressed as

(16) $${\text{SNR}}_{\text{res}}=\frac{P_{\text{I}}}{\sigma_{\text{n}}^{2}}{\left|h_{\text{I},\text{G}-\text{CRU}}\right|}^{2}+{\text{SNR}}_{\max\;}.$$

### 5.2. Relay Selection Stage

The problem of selecting a single (opportunistic) relay in a fully distributed manner was treated in [26, 27], while the method proposed there was extended to select multiple relays in [28]. However, due to the relatively low number of CRUs, that is, potential relays in the room, we apply the following simple centralized relay selection procedure.

The procedure is initiated after the transmission of the implant packet. At this time the CRUs (except for the G-CRU) send their received SNR to the G-CRU one after the other. The G-CRU then selects a number of *n* CRUs based on a selection criterion that takes into account the channel gain of both the implant-to-CRU*_i_* and the CRU*_i_*-to-G-CRU channels. (For details and examples, please refer to the next paragraph). At the end, the G-CRU broadcasts the outcome of the selection procedure to the CRUs. (Note that in both the present and the subsequent subsections, *i* = 2,…, *N* with the G-CRU being CRU_1_).

Concerning the relay selection criterion, we investigate two cases. For the criterion called *complex*, the G-CRU selects the CRUs with the highest value for SNR_I→CRU_*i*_→G−CRU_. This quantity is the received SNR for the implant-to-CRU_*i*_-to-G-CRU communication channel for the hypothetic case when all of the total relay power is allocated to CRU_*i*_ [29] and is expressed as

(17) SNRI→CRUi→G−CRU=Bi1+C  i  .

For the criterion named *simple*, the G-CRU simply selects the CRUs with the highest received SNR.

Based on (15), one can immediately find that selecting the nodes with the highest received SNR is the optimal relay selection criterion when *P*_CRU_ → ∞. On the other hand, since the expression of SNR_max _ for the finite *P*_CRU_ case (11) is fairly complicated and most probably cannot be “decoupled” as (15), the optimal relay selection criterion for this case remains an open research problem.

### 5.3. Power Allocation Stage

At the beginning of the power allocation procedure (taking place after the relay selection stage), the G-CRU calculates *g*(*n*_1_), which is then broadcasted to the relaying CRUs. Finally, the relays determine their transmission power level based on the value received [25].

### 5.4. Lifetime Gain

In this part, the lifetime gain to be evaluated in the performance analysis is defined.

As already mentioned above, in the case of the traditional, noncooperative communication approach, only a single receiver unit (SRU) is used in the immediate vicinity of the patient.

Though the SRU can be worn also on the patient's belt in practice, we assume an off-body SRU in our model for the following three reasons. Firstly, one of the aims of the MICS standard was to enable a communication distance measurable in meters [3]. Secondly, to the best of our knowledge, no in-body to on-body propagation model for the UHF band has been published in the literature yet. (Remark that, when worn on the belt or placed off-body close to the patient, the SRU is apparently in the near-field of the implant. For this reason, it is difficult to tell—without proper calculations or measurements—whether, at all, it is more advantageous to attach the SRU to the patient or it is more beneficial to install it off-body.) Thirdly, not wearing the SRU on the body all the time provides more comfort to the patient.

We are going to model the location of the SRU (**r**_SRU_) as if it were identical to that of the CRU that is found the closest to the implant in the statistical topology model introduced in Section 3, that is,

(18) $$r_{\text{SRU}}=r_{\arg\underset{i=1,...,N}{\min\;}|r_{i}-r|}.$$

In this way, both the relative proximity and the uncertain placement of the SRU are taken into account in our model. The resultant SNR of the implant packet when only a single receiver unit is employed is given by

(19) $${\text{SNR}}_{\text{res}}=\frac{P_{\text{I}}}{\sigma_{\text{n}}^{2}}{\left|h_{\text{I},\text{SRU}}\right|}^{2},$$

where *h*_I,SRU_ is the gain of the implant-to-SRU channel.

The reliability constraint we place on the communication link from the implant to the outside world is formulated as

(20) $$P\left({\text{SNR}}_{\text{res}}\left(P_{\text{I}}\right)<{\text{SNR}}_{\text{req}}\right)\leq P_{\text{out}}^{\max\;},$$

where SNR_req_ and *P*_out_^max^ are the required SNR and the maximum outage probability, respectively. In other words, the transmission power of the implant shall be high enough in order for the outage event of an unacceptably low resultant SNR to have a sufficiently low probability.

Finally, we define the lifetime gain as the relative difference in lifetime between the cooperative and noncooperative approaches. It is a reasonable assumption that the energy consumption of the implant is dominated by the transmission activity and that the energy consumption associated with the other activities such as reception or sensing can be neglected due to either their relatively low duty-cycle or power requirement. As a direct consequence of this, lifetime is assumed to be directly proportional to the reciprocal of the power consumed at transmission (*P*_t_) and, hence, the lifetime gain takes the form of

(21) $$G_{\text{lifetime}}=\frac{P_{\text{t}}\left(P_{\text{I}}^{\text{non-coop}}\right)}{P_{\text{t}}\left(P_{\text{I}}^{\text{coop}}\right)}-1.$$

Here *P*_I_^non-coop^ and *P*_I_^coop^ denote the minimum transmission power level of the implant that satisfies (20) for the noncooperative and cooperative communication schemes, respectively.

## 6. Performance Analysis

The model elaborated in the preceding sections is obviously analytically intractable generating the need for analytical approximations. However, in order to obtain controllably accurate results, the performance analysis is carried out numerically using Monte Carlo simulations.

The performances of the noncooperative and cooperative approaches are compared under the assumption of different room sizes. We consider a 3 × 3 m, a 5 × 5 m, and a 7 × 7 m room. (The corresponding room area (*A*) values are 9 m^2^, 25 m^2^, and 49 m^2^, resp.) In order for the distance of the patient and the closest CRU to have a mean of approximately 1 m, the spatial density of the CRUs (*ρ*) was set to 0.36 m^−2^, 0.34 m^−2^, and 0.31 m^−2^, respectively. (These values were determined by trial and error.) In terms of CRU antenna polarization, we investigate two cases. For the first one, CRUs are equipped with circularly polarized antennas, while for the second one, CRU antennas are linearly polarized with a uniformly random orientation.

**Figure 3:**
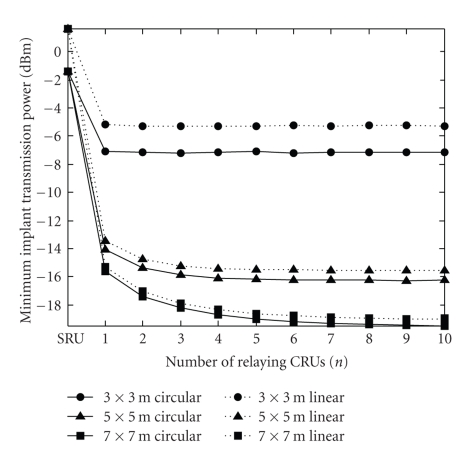
Minimum implant transmission power versus number of relaying CRUs (*n*) (*P*_CRU_ = ∞). The case of the traditional, noncooperative approach is also plotted and referred to as “SRU”. The different curves belong to different room sizes and CRU antenna polarizations (circular or linear).

Figure 3 describes the minimum implant transmission power (*P*_I_^coop^) as a function of the number of relaying CRUs (*n*) under the assumption of infinite total relay power (*P*_CRU_ = ∞). (The additional parameter values—not yet defined above—are listed in Table 1.) The minimum implant transmission power for the noncooperative approach (*P*_I_^non-coop^) is also plotted—at the tick labeled “SRU”—in the same diagram. (In case the value of variable *n* is higher than the number of nongateway CRUs in the room, i.e., *N* − 1, the actual number of relaying CRUs is limited to *N* − 1.) The diagram shows that the transmission power of the implant can considerably be reduced by employing the above described cooperative strategy. We can observe, in addition, that a slightly better performance can be obtained if the CRUs are supplied with circularly polarized antennas. Figure 3 also shows that the minimum implant transmission power decreases as the room size increases. Furthermore—while, for the two larger rooms, the performance improves as the number of relaying CRUs grows—in the case of the smallest room, applying more than one relaying CRUs does not result in an additional decrease in transmission power. Both of the latter phenomena are certainly due to the fact that the larger the rooms, the more CRUs are installed providing a higher degree of diversity.

**Figure 4:**
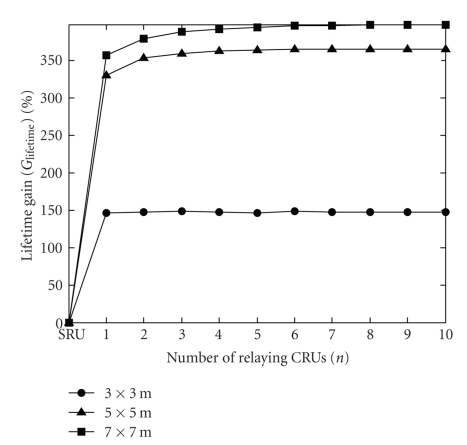
Lifetime gain (*G*_lifetime_) versus number of relaying CRUs (*n*) (*P*_CRU_ = ∞, circular CRU antenna polarization). The case of the traditional, noncooperative approach is also plotted and referred to as “SRU”. The different curves belong to different room sizes.

Figure 4 describes the lifetime gain (*G*_lifetime_) as a function of the number of relaying CRUs (*n*) for *P*_CRU_ = ∞ and for circularly polarized CRU antennas. The lifetime gain for the noncooperative approach—which is by definition equal to 0—is also plotted as a reference in the same diagram. It should be noted that the lifetime of the implant can significantly be prolonged by applying the cooperative communication approach described above. We also find that—though the minimum transmission power of the implant is approximately 3 dB lower for the 7 × 7 m room than for the 5 × 5 m room (Figure 3)—the difference in lifetime gain between the two larger rooms is not that pronounced. The reason for that is when the transmission power of the implant is around −15 dBm or lower, the power consumption of the implant (at transmission) is already dominated by the circuit power consumption (*P*_circ,t_) rather than the consumption of the RF PA (*η*_PA_^−1^ *P*_I_).

**Figure 5:**
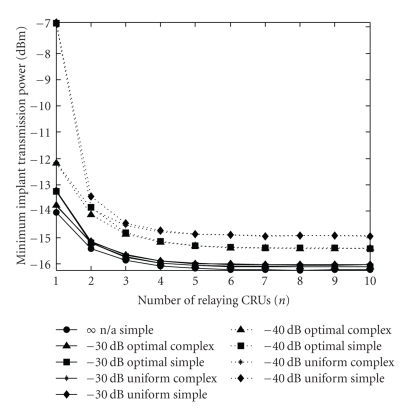
Minimum implant transmission power versus number of relaying CRUs (*n*) (*P*_CRU_ < ∞, circular CRU antenna polarization, 5 × 5 m room). The different curves belong to different *P*_CRU_ values, power allocation strategies (optimal or uniform) and relay selection criteria (complex or simple). The curve corresponding to *P*_CRU_ = ∞ is also plotted as a reference.

Figure 5 shows the minimum implant transmission power (*P*_I_^coop^) as a function of the number of relaying CRUs (*n*) for *P*_CRU_ < ∞, for circularly polarized CRU antennas and for a room size of 5 × 5 m. The total transmission power of the relaying CRUs (*P*_CRU_) power was set to −30 dB and −40 dB relative to *P*_circ,t_ (circuit power consumption) multiplied by the actual number of relaying CRUs. The simulations were performed under the assumption of different power allocation strategies (optimal or uniform) and relay selection criteria (complex or simple). The curve corresponding to *P*_CRU_ = ∞ is also plotted as a reference. Our first observation is that when the transmission power of the relaying CRUs is set to approximately −30 dB (1000 times) lower than *P*_circ,t_, the performance is practically identical to that of the system with *P*_CRU_ = ∞, no matter what power allocation strategy and relay selection criterion we choose. (This obvious and remarkable difference in the performance of the implant-to-CRU_*i*_ and CRU_*i*_-to-CRU_*j*_ communication channels is mainly due to the fact that the latter channels are not subject to the losses caused by the body tissues.) As a consequence, the power allocation stage can be omitted from the above described cooperative communication scheme, while in terms of relay selection, it is sufficient to use the simple criterion. On the other hand, when the transmission power of the relaying CRUs is set to approximately −40 dB relative to *P*_circ,t_, an apparent difference in performance is found for the different power allocation strategies, while the different relay selection criteria—except for *n* = 1,2—show practically the same performance. Nevertheless, in order for the choice of the power allocation strategy to have a considerable impact on the power consumption of the CRUs, the propagation conditions between the CRUs would need to be much more adverse than assumed in this study. (The same holds for the choice of the relay selection criterion concerning system performance.) Such conditions could perhaps occur if the room (or the part of the building) under investigation were—as an example—extremely densely furnished.

**Figure 6:**
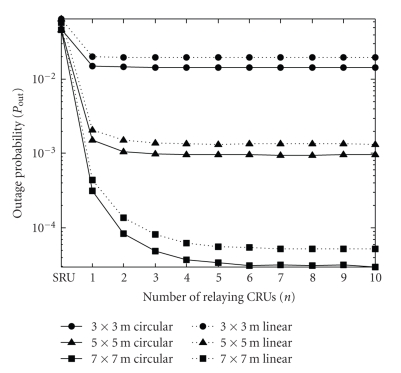
Outage probability (*P*_out_) versus number of relaying CRUs (*n*) (*P*_CRU_ = ∞, *P*_I_ = −9 dBm). The case of the traditional, noncooperative approach is also plotted and referred to as “SRU”. The different curves belong to different room sizes and CRU antenna polarizations (circular or linear).

Finally, Figure 6 describes the outage probability [*P*_out_, i.e., the left side of (20)] as a function of the number of relaying CRUs (*n*) for an implant transmission power of *P*_I_ = −9 dBm and for *P*_CRU_ = ∞. (The outage probability for the noncooperative approach is plotted at the tick labeled “SRU” in the same diagram.) The diagram shows that by employing the above cooperative strategy, the outage probability can considerably be reduced, that is, the reliability of the communication link between the implant and the outside world can greatly be improved. (The behavior of *P*_out_ as a function of variable *n*, the room size, and CRU antenna polarization are similar to the dependences observed for the minimum implant transmission power in Figure 3.)

## 7. Conclusions

We have shown in our investigations that the application of cooperative diversity is a promising approach to enhance system performance in medical implant communication systems in various aspects. Since it is a plausible assumption that the presence of the patient is limited to only a couple of rooms during the day, the reduction in transmission power that can be achieved may also result in an actual and considerable lifetime gain for the implant in practice. In addition to extending the lifetime of the implant, the above described collaborative procedure offers the alternative benefit of a more reliable, more spectrum efficient, more frequent, and more intensive data communication between the implant and the outside world. Finally, the above method provides more comfort to the patient as the SRU does not need to be worn on the belt all the time or transported upon changing location in the room.

The experimental verification of the results is not that obvious as one may think at first glance. The implantation requires an invasive intervention, and, for this reason, it should be preceded by a more extensive and accurate simulation work (e.g., FDTD simulations) even if the surgery is carried out—for instance—only on a (healthy) animal. One option is to employ patients with an implant already in their body. The problem with this alternative is that it demands the development of customized external devices that conform to both the proposed cooperative communication scheme and the specific implanted device in question.

The numerical results presented in the previous section were produced based on an approximate radio propagation model and, as a consequence, the exact figures should be considered with some caution. Nonetheless, as the order of magnitude of the transmission power levels calculated for the implant is believed to be correct, we hope that our study stimulates further and more detailed investigations of the subject in the interdisciplinary research community that is formed by communications technologists, physicians, and electromagnetic field theoreticians.

## Figures and Tables

**Table 1:** Simulation Parameters.

Parameter	Value	Unit
*η*	−31.1 [1]	dB
*η*_PA_	33.33 [18]	%
*P*_circ.,t_	−3/0.5 [16]	dBm/mW
SNR_req_	0	dB
*P*_out_^max^	10^−2^	—
Bandwidth	300 [2]	kHz
CRU noise figure	8 [3]	dB
*λ*	74.35 [2]	cm
Total CRU noise power over thermal noise power	20 [3]	dB

## References

[1] Hall PS, Hao Y (2006). *Antennas and Propagation for Body-Centric Wireless Communications*.

[5] http://www.biotronik.de/.

[6] http://www.medtronicconexus.com/.

[7] Johansson AJ Performance of a radio link between a base station and a medical implant utilising the mics standard.

[8] Johansson AJ (2004). *Wireless communication with medical implants: antennas and propagation*.

[9] Kailas A, Ingram M Transmit diversity for long-term implants.

[10] Laneman JN, Wornell GW (2003). Distributed space-time-coded protocols for exploiting cooperative diversity in wireless networks. *IEEE Transactions on Information Theory*.

[11] Sendonaris A, Erkip E, Aazhang B (2003). User cooperation diversity—part I: system description. *IEEE Transactions on Communications*.

[12] Yang G-Z (2006). *Body Sensor Networks*.

[13] Song L, Hatzinakos D (2006). Cooperative transmission in poisson distributed wireless sensor networks: protocol and outage probability. *IEEE Transactions on Wireless Communications*.

[14] Tekin A, Yuce MR, Shabani J, Wentai L A low-power FSK modulator/demodulator for an MICS band transceiver.

[15] Tanguay LF, Sawan M A fully-integrated 580 *μ*W ISM-band frequency synthesizer for implantable medical devices.

[16] Neihart NM, Harrison RR A low-power FM transmitter for use in neural recording applications.

[17] El-Desouki MM, Jamal Deen M, Haddara YM A low-power CMOS class-E power amplifier for biotelemetry applications.

[18] Abdelsayed SM, Deen MJ, Nikolova NK A fully integrated low-power CMOS power amplifier for biomedical applications.

[19] Picasso LB, Jansson P (2004). *Simulation and measurement of radio wave propagation in the 400 MHz MICS band*.

[20] Molisch AF (2005). *Wireless Communications*.

[21] Scanlon WG, Burns JB, Evans NE (2000). Radiowave propagation from a tissue-implanted source at 418 MHz and 916.5 MHz. *IEEE Transactions on Biomedical Engineering*.

[22] Johansson AJ, Karlsson A Wave-propagation from medical implants—influence of arm movements on the radiation pattern.

[23] Alomainy A, Hao Y, Yuan Y, Liu Y Modelling and characterisation of radio propagation from wireless implants at different frequencies.

[24] Laneman JN, Tse DNC, Wornell GW (2004). Cooperative diversity in wireless networks: efficient protocols and outage behavior. *IEEE Transactions on Information Theory*.

[25] Cui S, Xiao J-J, Goldsmith AJ, Luo Z-Q, Poor HV (2007). Estimation diversity and energy efficiency in distributed sensing. *IEEE Transactions on Signal Processing*.

[26] Bletsas A, Khisti A, Reed DP, Lippman A (2006). A simple cooperative diversity method based on network path selection. *IEEE Journal on Selected Areas in Communications*.

[27] Bletsas A, Lippman A, Reed DP A simple distributed method for relay selection in cooperative diversity wireless networks, based on reciprocity and channel measurements.

[28] Hegyi B, Levendovszky J Efficient, distributed, multiple-relay selection procedures for cooperative communications.

[29] Bletsas A, Shin H, Win MZ (2007). Outage optimality of opportunistic amplify-and-forward relaying. *IEEE Communications Letters*.

